# Mapping quantitative trait loci (QTLs) for fatty acid composition in an interspecific cross of oil palm

**DOI:** 10.1186/1471-2229-9-114

**Published:** 2009-08-26

**Authors:** Rajinder Singh, Soon G Tan, Jothi M Panandam, Rahimah Abdul Rahman, Leslie CL Ooi, Eng-Ti L Low, Mukesh Sharma, Johannes Jansen, Suan-Choo Cheah

**Affiliations:** 1 Advanced Biotechnology and Breeding Centre, Biology Division, Malaysian Palm Oil Board (MPOB), No. 6, Persiaran Institusi, Bandar Baru Bangi, 43000 Kajang, Selangor DE, Malaysia; 2 Department of Cell and Molecular Biology, Faculty of Biotechnology and Biomolecular Sciences, Universiti Putra Malaysia, 43400 UPM Serdang, Selangor, Malaysia; 3 Department of Animal Science, Faculty of Agriculture, Universiti Putra Malaysia, 43400 UPM Serdang, Selangor, Malaysia; 4 Research Department, United Plantations Berhad, Jenderata Estate, 36009, Teluk Intan, Perak, Malaysia; 5 Biometris, Wageningen University and Research Centre, P.O. Box 100, 6700 AC Wageningen, the Netherlands; 6 Asian Agri Group, Research & Development Centre, PO Box 35, Kebun Bahilang' Tebing Tinggi Deli 20600, North Sumatera, Indonesia; 7 Asiatic Centre for Genome Technology Sdn Bhd (ACGT), Lot L3-I-1, Enterprise 4, Technology Park Malaysia, 57000 Kuala Lumpur, Malaysia

## Abstract

**Background:**

Marker Assisted Selection (MAS) is well suited to a perennial crop like oil palm, in which the economic products are not produced until several years after planting. The use of DNA markers for selection in such crops can greatly reduce the number of breeding cycles needed. With the use of DNA markers, informed decisions can be made at the nursery stage, regarding which individuals should be retained as breeding stock, which are satisfactory for agricultural production, and which should be culled. The trait associated with oil quality, measured in terms of its fatty acid composition, is an important agronomic trait that can eventually be tracked using molecular markers. This will speed up the production of new and improved oil palm planting materials.

**Results:**

A map was constructed using AFLP, RFLP and SSR markers for an interspecific cross involving a Colombian *Elaeis oleifera *(UP1026) and a Nigerian *E. guinneensis *(T128). A framework map was generated for the male parent, T128, using *Joinmap *ver. 4.0. In the paternal (*E. guineensis*) map, 252 markers (199 AFLP, 38 RFLP and 15 SSR) could be ordered in 21 linkage groups (1815 cM). Interval mapping and multiple-QTL model (MQM) mapping (also known as composite interval mapping, CIM) were used to detect quantitative trait loci (QTLs) controlling oil quality (measured in terms of iodine value and fatty acid composition). At a 5% genome-wide significance threshold level, QTLs associated with iodine value (IV), myristic acid (C14:0), palmitic acid (C16:0), palmitoleic acid (C16:1), stearic acid (C18:0), oleic acid (C18:1) and linoleic acid (C18:2) content were detected. One genomic region on Group 1 appears to be influencing IV, C14:0, C16:0, C18:0 and C18:1 content. Significant QTL for C14:0, C16:1, C18:0 and C18:1 content was detected around the same locus on Group 15, thus revealing another major locus influencing fatty acid composition in oil palm. Additional QTL for C18:0 was detected on Group 3. A minor QTL for C18:2 was detected on Group 2.

**Conclusion:**

This study describes the first successful detection of QTLs for fatty acid composition in oil palm. These QTLs constitute useful tools for application in breeding programmes.

## Background

The oil palm is a perennial crop that belongs to the genus *Elaeis *and to the botanical family Palmae. Within the genus *Elaeis*, two species are distinguished, the economically important oil palm (*Elaeis guineensis*) originally native to Africa and the economically less important South American relative, *Elaeis oleifera *(which inherently has lower oil yield potential). The oil palm produces two distinct types of oil based on the fatty acid composition. The mesocarp of the fruit produces an oil (crude palm oil or CPO) which has a predominantly higher palmitic (C16:0) and oleic acid (C18:1) profile. In contrast, the endosperm (enclosed in a nut) produces oil (crude palm kernel oil or CPKO) in which the lauric fatty acids (C12:0) are predominant.

The main feature of the *E. oleifera *palm that distinguishes it morphologically from the commercial species *E. guineensis *is its procumbent trunk, distinctly smaller sized fruits and smaller canopy. Moreover, the angle of insertion of its leaflets is in a single plane as compared to a double plane for *E. guineensis *[1,2]. In *E. oleifera*, up to 65% of the fruits tend to be parthenocarpic [1] and have a much lower oil content [3]. As such, the oil yield of *E. oleifera *is much lower, with oil to bunch ratio of 5%, as compared to the *E. guineensis *(tenera) with oil to bunch ratio of more than 25% [4]. Nevertheless, *E. oleifera *possess certain important characteristics that are of significant interest to oil palm breeders. This includes the low annual stem height increment (between 5 and 10 cm per year as compared to between 45 to 65 cm per year for *E. guineensis*) [1,2]. The fatty acid composition of its CPO is especially of interest since its iodine value (IV, which is a measure of the degree of unsaturation of the oil) can be as high as 90 as compared to the average of 53 of *E. guineensis *[4]. The CPO derived from the *E. oleifera *oil has high levels of oleic and linoleic acid and lower levels of the palmitic acid and other saturated fatty acids, thus imparting a property quite akin to olive oil in composition. In South America, interest in the *E. oleifera *was driven by the fact that it shows resistance to bud rot disease [5].

In view of the apparent lack of variability for traits associated with high oil yield within *E. oleifera *and because *E. guineensis *has all the desired attributes for high oil yield, the only viable proposition (using conventional plant breeding approach) is to carry out interspecific hybridization between the two species. Fortunately, the *E. guineensis *and *E. oleifera *hybridize readily, producing fertile offspring in spite of their different areas of origin, which implies that they share a common ancestry before the two continents (Africa and South America) drifted apart some 110 million years ago. The fact that the two species can still hybridize to produce viable offspring itself suggests that the species isolation barrier is incomplete [1] despite the millions of years of separation.

The interspecific hybridization approach is viewed as a viable method to introgress the traits of interest i.e. namely higher oil unsaturation (to obtain a more liquid olein) [1,6]. This is a long term breeding strategy, with results obtained thus far showing that oil quality, taken as unsaturated fatty acid content, is better in the hybrids and in their backcrosses than in the commercial *E. guineensis *[1,7,8]. However, the conventional breeding approach is severely hampered by the fact that being a perennial crop, the oil palm has a long selection cycle of between 10 and 12 years [9]. Furthermore, it requires enormous resources in terms of land (usually one can only plant between 136 and 148 palms per hectare), labour and field management in breeding trials. The development of marker-assisted selection (MAS) techniques would greatly facilitate hybrid-breeding programmes as well as speed up the development of planting materials with an oil composition high in unsaturated fatty acids (especially oleic fatty acid). With MAS, selection can be carried out in segregating generations of interspecific hybrids and their backcrosses more discriminately using molecular markers linked to the specific fatty acids.

For the purpose of MAS, a number of DNA marker systems have been applied to genetic mapping in oil palm. Restriction Fragment Length Polymorphism (RFLP) markers from genomic libraries have been applied to oil palm linkage mapping [10]. This map harbouring 97 RFLP markers in 24 groups of two or more was generated using a selfed *guineensis *cross. Moretzsohn *et al*. [11] reported genetic linkage mapping for a single controlled cross of oil palm using random amplified polymorphic DNA (RAPD) markers and the pseudo-testcross mapping strategy described by Grattapaglia *et al*. [12]. More recently, Billotte *et al*. [13] reported a simple sequence repeat (SSR)-based high density linkage map for oil palm, involving a cross between a thin shelled *E. guineensis (tenera) *palm and a thick shelled *E. guineensis (dura) *palm. The map consisting of 255 SSR markers and 688 amplified fragment length polymorphism (AFLP) markers represents the first linkage map for oil palm to have 16 independent linkage groups corresponding to the haploid chromosome number of 16 in oil palm [14]. Mayes *et al*. [10], Moretzsohn *et al*. [11] and Billotte *et al*. [13] reported the identification of RFLP, RAPD and AFLP markers respectively, linked to the shell thickness locus, an important economic trait which exhibits monofactorial inheritance. However, most of the traits of economic interest in oil palm exhibit quantitative inheritance. In this area, Rance *et al*. [15], expanding on the genetic map developed by Mayes *et al*. [10], reported the detection of QTLs associated with vegetative and yield components of oil palm. The work reported above represents important developments in the application of MAS in oil palm breeding programmes. Despite the advances being made and the progress achieved in genetic mapping of oil palm, only a limited number of economically important traits have been tagged to date. Furthermore, none has been reported for fatty acid composition. This is probably because of the lower variability for most fatty acids within the *E. guineensis *populations.

In this study, we hoped to exploit the use of complementary DNA (cDNA) probes as RFLP markers for linkage map construction. The cDNA clones represent gene fragments that occur in the expressed regions of the genome. Their identity can be determined via sequencing and such sequences are known as expressed sequence tags (ESTs). The usefulness of ESTs as markers has been demonstrated in several plant species [16,17]. ESTs help to map known genes apart from providing anchor probes for comparative mapping. Furthermore, mapping ESTs closely linked to or co-segregating with a trait allows the gene for that trait to be identified by the candidate gene approach. This could eventually expedite the application of MAS in oil palm breeding programmes.

The strategy adopted in this research was to capitalize on the differences between the two species of oil palm and use an interspecific hybrid for the analysis of QTLs associated with palm oil fatty acid composition. This study employed both dominant (AFLP) and co-dominant (RFLP and SSR) markers to generate a linkage map. The map was subsequently used to locate QTLs associated with the fatty acid composition.

## Results

### Marker Screening

A total of 413 polymorphic AFLP loci were scored in the progeny by using the 67 AFLP primer pairs (Table 1). Generally, for the majority of the segregating markers scored, 405 (98%) were in the pseudo-testcross configuration where either the male parent was heterozygous, and the fragment was absent in the female parent (type b profile) or vice versa (type a profile) (Table 2).

**Table 1:** Summary of RFLP, SSR and AFLP analysis of the interspecific hybrid mapping population

Type of markers	No. of probes/primer pairs evaluated	No. of informative probes/primer pairs	No. of polymorphic loci identified	No. of markers showing 3:1 segregation	No. of markers showing 1:1 segregation in the gametes of		No. of markers meeting goodness-of-fit to 1:1, 1:1:1:1 or 3:1 ratio
					T128	UP1026	
AFLP	67	67	413	8	360	45	323
RFLP	289	71	76*	-	71	5	63
SSR	56	19	23**	-	22^#^	1	18

Total			512	8	453	51	404

* Five RFLP markers detected two loci each

** Four SSR primers detected two loci each

^# ^Three of the SSR markers showing 1:1:1:1 segregation ratio (type f and g, Table 2) were grouped here for simplicity of presentation

**Table 2:** Parent and progeny phenotypes for AFLP, RFLP and SSR markers in the mapping population

Loci defined by		Parent Genotypes		Progeny Genotypes				Expected Segregation ratio	No of Segregating Markers^a^		
	Type	Oleifera	Guineensis	1	2	3	4		AFLP	RFLP	SSR
Single band	a	^_^		^_^		^_^		1:1	45	4	-
	b		^_^	^_^			^_^	1:1	360	47	10
	c	^_^	^_^	^_^	^_^	^_^		3:1	8	-	-
											
Two alleles	d	^_^		^_^		^_^		1:1	-	1	1
		^_^			^_^		^_^				

	e		^_^	^_^		^_^		1:1	-	24	9
			^_^		^_^		^_^				
											
Three alleles	f	^_^		^_^			^_^	1:1:1:1	-	-	1
		^_^	^_^	^_^	^_^	^_^					
			^_^			^_^	^_^				
											
Four alleles	g	^_^		^_^		^_^		1:1:1:1	-	-	2
		^_^			^_^		^_^				
			^_^	^_^			^_^				
			^_^		^_^	^_^					

^a ^Refers to the number of markers having each segregation pattern among the progeny of the UP1026 (*E. oleifera*) × T128 (*E. guineensis*) interspecific cross

A total of 289 cDNA probes from various cDNA libraries were tested for their ability to detect segregation in the progeny using the RFLP approach. Of the 289 probes screened, 71 (24.6%) showed polymorphisms with at least one restriction enzyme, 167 (58%) were monomorphic and 51 (17.7%) gave no clear banding pattern. The percentage of polymorphic probes identified (24.6%) was similar to the rate of 25% polymorphic RFLP probes (from genomic library) reported previously by Mayes *et al*. [10] for oil palm. Out of the 71 RFLP probes showing polymorphism, 66 (93%) were inherited from the male *E. guineensis *parent. Five of these 66 probes revealed two polymorphic loci each, giving a total of 71 polymorphic loci (Tables 1 and 2). The RFLP probes used in this study appeared to have mainly scanned the homozygous regions of the *E. oleifera *parental palm that were not segregating in the mapping progeny, thus reducing the number of polymorphic probes revealed.

Among the 33 SSR primer pairs developed in the course of this study, nine were informative and segregating in the mapping population. Of the 20 single-locus SSR primer pairs reported by Billotte *et al*. [18], seven segregated in the mapping population. Six segregated in the male *E. guineensis *parental gametes only, while one segregated in the female *E. oleifera *gametes. Three of the five EST-SSRs tested (CNH0887, CNH1537 and EAP3339) showed polymorphism in the mapping population. All three informative primer pairs segregated only in the male parent *E. guineensis *gametes. Four of the informative SSR primers segregating in the male gametes revealed two loci each (Table 1). Information on the informative SSR primer pairs is provided in Tables 3 and 4.

**Table 3:** Microsatellite loci developed in the course of this study

*No*.	*Locus name*	Accession Number*
1	P1A0	9722519
2	P1T6b	9722520
3	P1T12b	9722521
4	P4T8	9722522
5	P4T10	9722523
6	P4T12a	9722524
7	P4T20b	9722525
8	P1014a	9722526
9	P201b	9722527
10	CNH0887 (EST-SSR)	9722528
11	CNHI537 (EST-SSR)	9722529
12	EAP339 (EST-SSR)	9722530

* *GenBank *(NCBI Probe Database)

**Table 4:** Microsatellite locus reported by Billotte *et al*. [18]

*No*.	*SSR Locus*	*EMBL Accession Number*
1	mEgCIR0008	AJ271625
2	mEgCIR0009	AJ271633
3	mEgCIR0018	AJ271634
4	mEgCIR0046	AJ271635
5	mEgCIR0067	AJ271636
6	mEgCIR0377	AJ271936
7	mEgCIR1772	AJ271937

Of the 512 (413 AFLP, 76 RFLP and 23 SSR) markers identified segregating in the mapping population, 453 (360 AFLP, 71 RFLP and 22 SSR) were segregating in the gametes of the male parent, Nigerian *E. guineensis *and 51 (9.9%) were segregating in the gametes of the female parent, the Colombian *E. oleifera *(Table 1). This indicated that the male *E. guineensis *parent is more heterozygous than the female parent, *E. oleifera*. As such, sufficient markers could only be generated to enable development of a genetic linkage map for the male parent. It is therefore concluded that it would be more appropriate to analyze this cross as a "one-way pseudo-testcross" in which the male, *E. guineensis *is considered to be the heterozygous parent and the Colombian *E. oleifera*, the homozygous parent.

### Linkage analysis

Only markers showing "Type b, e, f and g" profiles (Table 2) were used for linkage analysis. Markers showing "Type c" profile with a 3:1 segregation ratio (Table 2) were not employed as the recombination frequencies obtained with such markers are typically inaccurate [19]. In the initial attempt, 453 markers were shortlisted to generate a linkage map for the male T128 parent. Fourteen markers had to be removed from the analysis as they showed very significant distortion (P < 0.0001). In addition, 34 markers with more than 12 missing data points were also removed. Finally, 405 markers were used for map construction. Both the independence LOD and recombination frequency methods agreed with respect to the grouping of markers in the linkage groups. However, 15 of the markers (11 AFLP, three RFLP and one SSR) remained unlinked. These unlinked markers could be sampling parts of the genome where there are few other markers, in which case they would be very valuable in the future [20].

**Figure 1:**
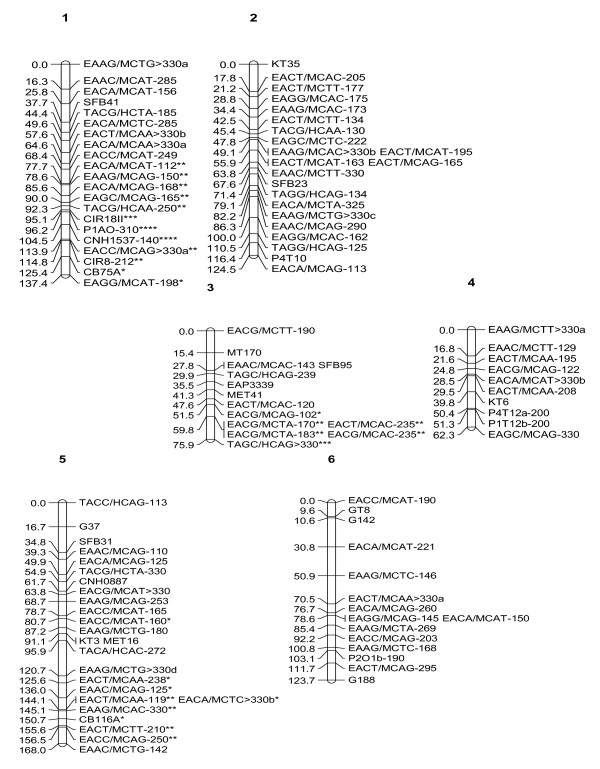
**Combined AFLP, SSR and RFLP Map of interspecific hybrid (Palm T128) (Linkage Groups 1–6)**. Single asterisk: skewed marker at P < 0.1; double asterisk: skewed marker at P < 0.05; three asterisks: skewed marker at P < 0.01; four asterisks: skewed marker at P < 0.005; five asterisks: skewed marker at P < 0.001; six asterisks: skewed marker at P < 0.0005.

**Figure 2:**
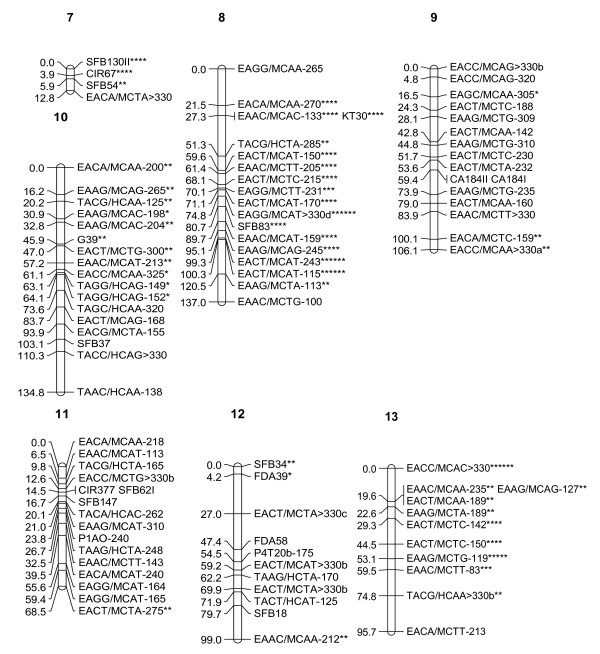
**Combined AFLP, SSR and RFLP Map of interspecific hybrid (Palm T128) (Linkage Groups 7–13)**. Single asterisk: skewed marker at P < 0.1; double asterisk: skewed marker at P < 0.05; three asterisks: skewed marker at P < 0.01; four asterisks: skewed marker at P < 0.005; five asterisks: skewed marker at P < 0.001; six asterisks: skewed marker at P < 0.0005.

**Figure 3:**
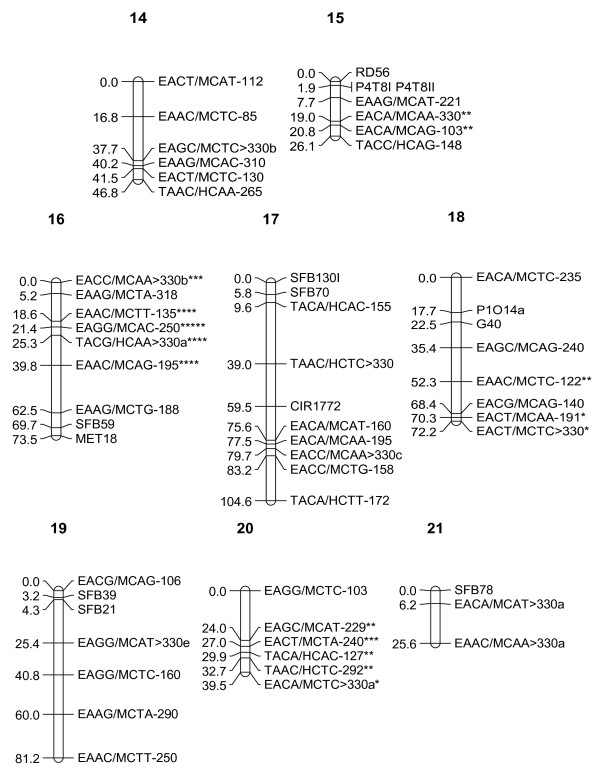
**Combined AFLP, SSR and RFLP Map of interspecific hybrid (Palm T128) (Linkage Groups 14–21)**. Single asterisk: skewed marker at P < 0.1; double asterisk: skewed marker at P < 0.05; three asterisks: skewed marker at P < 0.01; four asterisks: skewed marker at P < 0.005; five asterisks: skewed marker at P < 0.001; six asterisks: skewed marker at P < 0.0005.

In the initial map constructed, markers of two linkage groups (Groups 4 and 9) exhibited irregular patterns. In order to improve the map order, the total number of recombinations for each palm across linkage groups was evaluated. Out of the 118 palms used in the analysis, eight palms with relatively high recombination frequencies were identified. These eight palms were then removed from the analysis and map construction was repeated for all groups as before using the remaining 110 palms and the 453 markers that were shortlisted. In the second attempt, similarly, 14 markers had to be removed from the analysis as they showed very significant distortion (P < 0.0001). In addition, 36 markers with more than 12 missing data points had to be removed and hence 403 markers were finally used for map construction. The same 15 markers (11 AFLP, three RFLP and one SSR) that were unlinked in the previous attempt remained unlinked in this effort. The new map order was generally similar to the order produced previously and the "plausible position analysis" showed that marker order of all groups showed a regular pattern and all markers were indeed located at their "best estimated position". A graphical representation of the genetic linkage map obtained is shown in Figures 1, 2 and 3. In total, 252 markers (199 AFLP, 38 RFLP and 15 SSR) mapped in 21 linkage groups. The average number of markers per linkage group was 12. The total genetic distance covered by the markers was 1815 cM, with an average interval of 7 cM between adjacent markers. The map distance of the tenera T128 parental palm was close to the tenera map distance of 1,597 cM reported by Billotte *et al*. [13]. Excluding the two smallest groups (7 and 21) which had three and four markers respectively, the length of individual linkage groups varied from 26.1 cM to 168 cM, with an average of 94 cM. The average length of the linkage groups is close to the expected size of 100–150 cM found in most agricultural crops [19].

The markers were well distributed over all the 21 linkage groups. There was only one interval of 30 cM in Group 17. There were no gaps larger than 25 cM in any of the other groups. This indicates that the map is relatively homogeneous with regards to marker distribution and will be useful for tagging traits of economic interest for the purpose of marker-assisted selection.

Of the 71 RFLP loci used for linkage analysis, 38 were successfully mapped. The 38 RFLP loci were generated from 37 independent cDNA probes (Table 5). The RFLP markers were generally well distributed throughout the linkage groups. There were certain instances (e.g. Groups 17 and 19) where two RFLP markers were not interrupted by AFLP loci, which in fact tended to flank the RFLP markers. However, there were many regions where both marker systems intermingled and as such, probably do not at this stage represent distinct regions. Twenty-four of the RFLP sequences had significant similarity with *GenBank *accessions, particularly to genes from rice and *Arabidopsis *(Table 5). However, five of these matched with unknown or hypothetical proteins. The location of some putative genes (namely, class III peroxidase, embryo specific protein, profilin, pectinesterase, chitinase, class 3 alcohol dehyrogenase, histone H2B, metallothionein, ribosomal protein S26, actin depolymerizing factor and chrosimate synthase) were determined on the present linkage map.

**Table 5:** List of RFLP loci mapped, GenBank (dbEST database) accession number and gene identity

*No*.	*Probe*	*Linkage Group*	*Accession No*	Putative Gene ID#
1	SFB41	1	GH159190	No Hit
2	CB75A	1	GH159164	class III peroxidase (*Oryza sativa*)
3	KT35	2	GH159177	hypothetical protein (*Oryza sativa*)
4	SFB23	2	GH159185	No Hit
5	MT170	3	GH159181	No Hit
6	SFB95	3	GH159197	type 1 KH domain containing protein (*Populus tremula*)
7	MET41	3	GH159180	putative embryo specific protein (*Oryza sativa*)
8	KT6	4	GH159175	No Hit
9	G37	5	GH159168	No Hit
10	SFB31	5	GH159186	profilin (*Cocos nucifera*)
11	KT3	5	GH159174	No Hit
12	MET16	5	GH159178	No Hit
13	CB116A	5	GH159165	No Hit
14	GT8	6	GH159173	No Hit
15	G142	6	GH159171	No Hit (same as GT8)
16	G188	6	GH159172	stress responsive protein (*Triticium aestivium)*
17*	SFB130	7 & 17	GH159198	No Hit
18	SFB54	7	GH159191	pectinesterase family protein (*Arabidopsis thaliana*)
19	KT30	8	GH159176	chitinase (*Brassica rapa*)
20	SFB83	8	GH159196	unknown (*Populus trichocarpa)*
21*	CA184	9	GH159163	D6-type cyclin (*Populustrichocarpa)*
22	G39	10	GH159169	rab-type small GTP-binding protein (*Cicer arietinum*)
23	SFB37	10	GH159188	class 3 alcohol dehyrogenase (*Oryza sativa*)
24	SFB62	11	GH159193	eukaryotic translation initiation factor (*Arabidopsis thaliana*)
25	SFB147	11	GH159199	histone H2B, putative (*Arabidopsis thaliana*)
26	SFB34	12	GH159187	PVR3-like protein (*Ananas comosus*)
27	FDA39	12	GH159166	early-responsive to dehydration protein-related (*Arabidopsis thaliana*)
28	FDA58	12	GH159167	hyphothetical protein Os1_002257 (*Oryza sativa*)
29	SFB18	12	GH159183	hypothetical protein (*Vitis vinifera*)
30	RD56	15	GH159182	hypothetical protein (*Oryza sativa*)
31	SFB59	16	GH159192	pectinesterase inhibitor (*Medicago truncatula*)
32	MET18	16	GH159179	metallothionein-like protein (*Elaeis guineensis*)
33	SFB70	17	GH159194	ribosomal protein S26 (*Pisum sativum*)
34	G40	18	GH159170	actin depolymerizing factor (*Elaeis guineensis*)
35	SFB39	19	GH159189	No Hit
36	SFB21	19	GH159184	No Hit
37	SFB78	21	GH159195	chrosimate synthase (*Oryza sativa*)

# Putative Gene Identity was inferred from homology search using BLASTX.

* The RFLP markers concerned detected more than one segregating loci

Fifteen of the 22 SSR loci segregating in the male parent gametes were successfully mapped. Due to the low number of SSR markers employed, only ten of the groups had at least one SSR marker each. Nevertheless, the presence of SSR markers in these groups together with the RFLP markers makes it more convenient for genetic map integration or comparison. Development of additional SSRs from the existing ESTs collection is in progress, and it is anticipated that the EST-SSRs will assist with map saturation in the future.

The proportion of markers exhibiting distorted segregation ratio in this study was about 21% (Table 1). This was slightly higher than that reported for oil palm previously (less than 10%) [13] and other species, such as *Eucalyptus *(15%) [20] and apricot (17% for AFLP markers) [21]. However, the segregation distortion was much lower than those observed for roses (27%) [22] and coffee (30%) [23]. Nevertheless, 79% of the markers (Table 1) segregated in the expected ratios, indicating that a majority of the markers were inherited in a stable Mendelian manner. Groups 7, 8 and 13 in particular had a large percentage of distorted markers.

### Quantitative traits

A major objective of this study is to map QTLs associated with iodine value (IV) and fatty acid composition (FAC) in oil palm. Generally, all of the traits showed a pattern of continuous distribution around the mean, although some traits did not follow a perfect normal distribution (data not shown). The frequency distribution of IV, C16:0, C18:0, C18:1, C18:2 and C18:3 did not differ significantly from normality. This agrees with the co-dominant theory of inheritance for the fatty acids as proposed by Ong *et al*. [24]. However, the frequency distribution of C14:0 and C16:1 showed deviation from normality. Deviation of a trait from a perfect normal distribution has been observed in QTL analysis experiments [25].

The correlation coefficients between the various traits and their values were computed and provided in Table 6. As expected, the IV content is positively correlated with the unsaturated fatty acids C18:1 and C18:2. The results also indicate that the saturated fatty acids C14:0 and C16:0 are negatively correlated with IV, C18:1 and C18:2. The results obtained here are as anticipated and similar to those reported previously [26,27]. However, C18:0 showed no significant correlation to C16:0 and C18:1. Weak correlation between C16:0 and C18:0 has also been reported previously for rapeseed [28]. Nevertheless, Perez-Vich *et al*. [29] had reported that the C18:0 and C18:1 contents were negatively correlated in sunflower. The lack of correlation of C18:0 to C18:1 could be due to the low levels of inherent C18:0 in oil palm including the interspecific hybrids.

**Table 6:** Correlation between fatty acids (n = 81) in F_1 _progeny

	*IV*	*C14:0*	*C16:0*	*C16:1*	*C18:0*	*C18:1*	*C18:2*
IV							
C14:0	-0.679**						
C16:0	-0.879**	0.716**					
C16:1	-0.169	0.278*	0.186				
C18:0	-0.143	-0.107	0.062	-0.734**			
C18:1	0.733**	-0.646**	-0.941**	-0.219	-0.33		
C18:2	0.517**	-0.301**	-0.199	-0.044	-0.123	-0.111	
C18:3	0.202	0.059	-0.175	0.316**	-0.266	0.035	0.281*

Note: Correlation carried out using Pearson Correlation test implemented via the SPSS software package.

** Correlation is significant at the 0.01 level (2-tailed)

* Correlation is significant at the 0.05 level (2-tailed)

### QTL analysis

**Figure 4:**
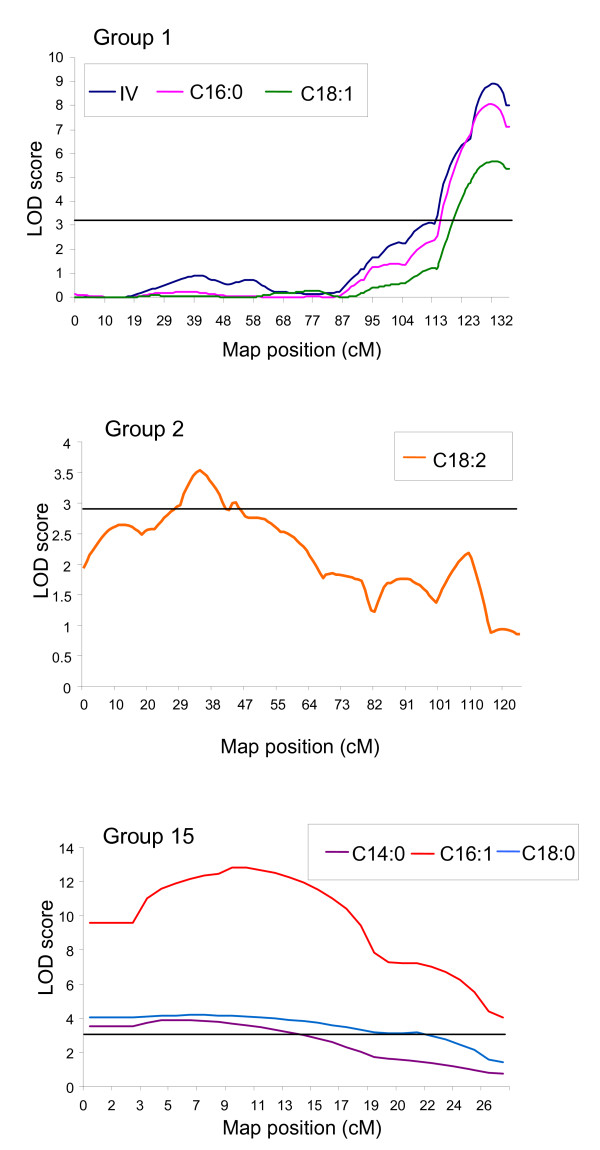
**QTL graphs for IV and the various fatty acid compositions on Groups 1, 2 and 15**. Results from the Interval Mapping approach. Horizontal line indicates the 95% significant threshold value for declaring a QTL.

At a genomic wide significant threshold of P < 0.01 and P < 0.05, significant QTLs were detected for IV (Group 1), C14:0 (Groups 8 and 15), C16:0 (Group 1), C16:1 (Group 15), C18:0 (Group 15), C18:1 (Group 1) and C18:2 (Group 2) using the interval mapping approach (Table 7). Significant QTLs were not detected for C18:3. The LOD score profiles obtained are shown in Figure 4.

**Table 7:** QTLs for IV and fatty acid composition found to be significant at the empirical genome wide mapping threshold (Interval Mapping)

Trait	Genome wide significant threshold level		Group	LOD Peak	Position of LOD peak (cM)	Left – Right Locus ^a^	% variance explained
						
	P < 0.05	P < 0.01					
IV	3.0	3.9	1	8.90	132.4	CB75A - EAGG/MCAT-198	46.3
C14:0	3.0	3.4	8	3.96	24.5	EACA/MCAA-270 - EAAC/MCAC-133	23.6
			15	3.92	4.8	P4T8 - EAAG/MCAT- 221	22.3
C16:0	3.1	4.0	1	8.06	132.4	CB75A - EAGG/MCAT-198	42.9
C16:1	3.1	3.7	15	12.8	7.7	P4T8 - EAAG/MCAT-221	56.6
C18:0	3.0	3.6	15	4.18	6.9	P4T8 - EAAG/MCAT-221	22.5
C18:1	3.0	3.8	1	5.69	133.4	CB75A - EAGG/MCAT-198	32.5
C18:2	2.9	3.5	2	3.54	34.4	EAGG/MCAC-175 - EAAG/MCAC-173	19.7

^a ^Loci flanking the likelihood peak of a QTL

In the subsequent multiple-QTL model (MQM) analysis, the significant QTLs for IV, C16:0 and C18:1 were maintained on Group 1. However, additional QTLs for C14:0, and C18:0 were also revealed on Group 1 (Table 8). All five QTLs showed similar shaped LOD profiles suggesting that the same QTL is influencing the five traits. The QTLs mapped on Group 1 for IV, C16:0 and C18:1 explain a significant proportion of the variation observed for the traits, that is 46.3% for IV, 44.4% for C16:0 and 33.1% for C18:1. The variation explained for C14:0 and C18:0 on Group 1 was 13.1% and 17.2% respectively, indicating that it was a minor QTL influencing these two traits. The QTL for unsaturation (C18:1 and IV) had an opposite effect to the QTL for saturated fatty acids (C16:0 and C18:0), suggesting that the alleles at this QTL locus affect the saturated and unsaturated fatty acids differently.

**Table 8:** QTLs for IV and fatty acid composition found to be significant using MQM mapping and Kruskal-Wallis analysis

*Trait*	*Co-Factor*	*Group*	*LOD* *Peak*	*Position* *of LOD peak* *(cM)*	*Left – Right Locus*^a^	*% variance* *explained*	*Kruskal-Wallis* *test (P)*
IV	EAGG/MCAT-198	1	9.55	132.4	CB75A^(-) ^- EAGG/MCAT-198^(-)^	46.3	0.0001 0.0001

C14:0	EAAC/MCAC-133	15	5.30	4.8	P4T8^(-) ^- EAAG/MCAT-221^(-)^	20.5	0.0001 0.0001
	& EAAG/MCAT-221	1	4.63	137.4	CB75A^(+) ^- EAGG/MCAT-198^(+)^	13.1	0.010 0.005

C16:0	EAGG/MCAT-198	1	8.92	132.4	CB75A^(+) ^- EAGG/MCAT-198^(+)^	44.4	0.0001 0.0001

C16:1	EAAG/MCAT-221	15	13.26	7.7	P4T8^(-) ^- EAAG/MCAT-221^(-)^	55.8	0.0001 0.0001

C18:0	EAAG/MCAT-221	15	5.10	6.9	P4T8^(+) ^- EAAG/MCAT-221^(+)^	23.2	0.0001 0.0005
		1	3.79	132.4	CB75A^(+) ^- EAGG/MCAT-198^(+)^	17.2	0.0050 0.0005
		3	3.24	75.9	TAGC/HCAG > 330^(+) ^-	16.3	0.005
					-		

C18:1	EAGG/MCAT-198	1	6.59	133.4	CB75A^(-) ^- EAGG/MCAT-198^(-)^	33.1	0.0001 0.0001
		15	3.04	7.7	P4T8^(+) ^- EAAG/MCAT-221^(+)^	12.8	0.010 0.010

C18:2	EAAG/MCAC-173	2	3.54	34.4	EAGG/MCAC175^(+)^- EAAG/MCAC-173^(+)^	19.7	0.0010 0.0005

^(+) ^The mean value of the quantitative trait for palms having the locus was higher compared to palms not having the locus

^(-) ^The mean value of the quantitative trait for palms having the locus was lower compared to palms not having the locus

The above analysis was possible as markers linked to the QTLs were in the pseudo-testcross configuration (type b profile, Table 2)

^a ^Loci flanking the likelihood peak of a QTL

P: Significance level

Significant QTLs for C14:0 and C18:0 were located on Group 15, which explained 20.5% and 23.2% of the variation respectively. Another major QTL located on Group 15 was that for C16:1, which explained 55.8% of the variation. A minor QTL for C18:1 was also located around the same region on Group 15 (revealed by MQM analysis), explaining about 12.8% of the variation respectively. The LOD profiles of the QTLs were also very similar (Figure 4, Table 8), indicating that the same QTL is influencing the traits concerned on Group 15. In contrast to what was observed in Group 1, the minor QTL for C18:1 on Group 15 was in the same direction with C18:0. Similar results were also observed by Zhao *et al*. [28] for rapeseed and could be an indication of a pleiotropic effect of a single QTL.

MQM analysis revealed a third minor QTL on Group 3 for C18:0. The minor QTL detected for C14:0 on Group 8 through Interval Mapping was found to be not significant in the MQM analysis, and as such, was not considered as a locus influencing C14:0 in this study. With respect to C18:2, only a single QTL was detected on Group 2 (both in Interval Mapping and MQM analysis), which explained about 19.7% of the variation observed.

The rank sum test of Kruskal-Wallis was subsequently used to confirm whether the individual markers linked to the QTLs were actually significant. The Kruskal-Wallis test is regarded as the non-parametric equivalent to the one-way analysis of variance [30], and the results are summarized in Table 8. For all traits, the markers flanking the QTL were also significant (P < 0.05) for the presence of a segregating QTL in the Kruskal-Wallis test. The Kruskal-Wallis test provides further confirmation of the marker-trait association, and indicates that the results of the QTL analysis were not influenced by segregation distortion or non-normal distribution of certain traits (14:0 and C16:1).

### Segregation of markers associated with QTLs

This study also correlated the actual segregation of RFLP and SSR markers (closest to the QTL peak) and the traits of interest in the mapping population. The RFLP and SSR markers were chosen, as they are practical for application in plant breeding and had significant LOD scores for the traits concerned. Since the pseudo-testcross strategy was used in map construction, palms in the mapping population were separated as either having the band present ("ab") or absent ("aa") for a particular marker associated with the QTL. The trait values were averaged and compared between palms with the "aa" and "ab" genotypes. The results obtained are summarized in Table 9. As is shown for the RFLP marker CB75A, there was a significant difference for IV between palms having the "aa" and "ab" genotypes. The absence of the CB75A band (aa) resulted in high levels of IV, in other words, high levels of unsaturation of the oil. Similar results were observed for C18:1. The RFLP probe CB75A was also associated with the QTL for C16:0 (palmitic acid). In a similar analysis, there were significant differences in the C16:0 content between palms having the "ab" and "aa" genotypes. In this case, the presence of the CB75A band is correlated with a higher level of the saturated fatty acid C16:0. The results are interesting as the presence of the CB75A band points to a higher level of saturated fatty acid (C16:0), lower levels of unsaturation (lower IV reading) and vice versa, in this particular mapping population. The sequence of the RFLP probe CB75A was however not associated with any of the genes in the fatty acid pathway.

**Table 9:** QTL effects expressed as differences between marker genotype classes for specific traits

*Trait*	*Marker*	*Genotype*	*Mean ± SE*
IV	CB75A	aa	73.26 ± 0.45^a^
		ab	69.59 ± 0.36^b^

C16:0	CB75A	aa	27.01 ± 0.40^a^
		ab	30.79 ± 0.39^b^

C16:1	P4T8	aa	0.52 ± 0.02^a^
		ab	0.34 ± 0.01^b^

C18:0	P4T8	aa	1.91 ± 0.04^a^
		ab	2.19 ± 0.05^b^

C18:1	CB75A	aa	55.15 ± 0.92^a^
		ab	52.50 ± 0.41^b^

Means of the genotypes "aa" and "ab" were compared using the independent t test via the SPSS statistical package. The means of the different genotypes for the markers associated with each trait were found to be significantly different

aa: band absent

ab: band present

In a similar way, the traits C16:1 and C18:0 were negatively correlated and the QTLs overlapped in the same position on Group 15. The presence of the SSR allele, P4T8 (band size 245 bp), which is located about 6 cM from the estimated position of the QTL, resulted in high levels of C18:0 but reduced levels of C16:1.

## Discussion

The FACs of palm oil and palm kernel oil render these oils applicable to both edible and non-edible uses. However, to venture into new markets, at least in the Malaysian palm oil industry, the focus is to change the oil towards a higher unsaturated FAC content particularly oleic acid (C18:1), at the expense of saturated fatty acids such as palmitic acid (C16:0) [31]. An oil with such properties has the potential to open up wider markets for palm oil in the salad oil sector, especially in the cooler climatic regions of the world where currently rapeseed, sunflower and soyabean are preferred [1]. In addition, it is envisaged that such an oil could be industrially useful for producing chemical derivatives, which could serve as alternatives to petrochemical feedstock [31] as well as be a potentially cheaper feedstock for production of biofuels by virtue of having a lower cloud point. The ultimate objective is to try to produce breeding lines that can produce oil with IV content of above 72, palmitic acid content of below 25% and oleic acid content of 60%, without sacrificing the palm oil yield per unit area. This will ensure that maximum benefit could be achieved from diversifying away from the present commercial planting material that has a higher saturated fatty acid profile and into a more liquid oil without sacrificing the inherent high oil yield potential of the crop [1,26].

Two approaches are being taken to achieve this objective: i) genetic engineering of oil palm [31,32] and ii) using the more conventional breeding approach of interspecific hybrid breeding. The work carried out in this study was also intended to develop probes to help expedite the latter approach, which is not complicated by issues of bio-safety and bioethics. The mapping population chosen for this purpose met two important criteria; segregating for the trait of interest and is relevant in the long term breeding scheme or strategy to improve the oil quality trait. Although the female parent (*E. oleifera*) was mostly homozygous for the loci analyzed, the male parent, *E. guineensis *fortunately was highly heterozygous, hence contributing to a significant level of genetic variability that was exploited for QTL analysis. In fact, it has been reported that the range of fatty acid composition observed in Nigerian based materials such as the male parent palm T128 used in this study, extends beyond that of the breeding materials currently in use [33]. This suggests that the Nigerian based *E. guineensis *materials are more suited for breeding oil palms with improved fatty acid composition [33]. The variation captured in the male parent *palm *T128 can also prove useful for selection within *E. guineensis*, which can directly affect desirable changes in fatty acid composition in hybrids created subsequently. Nevertheless, it is also acknowledged that to fully exploit the value of oil palm interspecific hybrids and to capture the variation between the two parents, backcross populations have to be analyzed in the future.

The genetic map constructed had an excess of linkage groups in relation to the haploid chromosome number despite the relatively high number of markers used. Failure to obtain the basic chromosome number despite applying high numbers of markers has also been reported for other species [34,35]. The reason for this could perhaps be due to the relatively small sample size of the F_1 _progeny used in this study. Another possible explanation is the lack of polymorphic markers in particular chromosomal regions, which could be due to the marker systems being employed and/or presence of large homozygous regions in the genome of the female *E. oleifera *parental palm used to create the interspecific hybrid population used in this study. Furthermore, it has to be stressed that very strict criteria were used to carry out map construction in this study. Only markers that fit extremely well in a linkage group were retained. Markers that caused even a slight friction were discarded in order not to compromise the subsequent QTL analysis. This also explains why only 252 markers (56%) were successfully ordered in the genetic map. The genetic map reported in this study depicts the mapping of expressed genes. The sequences of the RFLP markers mapped in this study have been submitted to *GenBank*. Since the RFLP markers were well distributed across the linkage groups, they can be used as potential anchor markers for integration or comparison of maps of different populations. As the oil palm EST database is growing rapidly [36,37], additional probes either as RFLP markers or EST-SSR markers will be placed on the genetic map concerned. More importantly, the growing oil palm EST database will allow the selective mapping of genes associated with the fatty acid composition pathway. The use of allele specific markers linked to genes underlying the synthesis of seed oils has been demonstrated in *Brassica *[38].

In this study, 11 QTLs were detected for IV and the six components of the fatty acid composition (C14:0. C16:0, C16:1, C18:0, C18:1 and C18:2) in four different linkage groups. For C18:1, two QTLs were detected, one major QTL in Group 1 and a minor QTL in Group 15, which collectively explained 45.9% of the total phenotypic variation. Two QTLs were detected for C14:0 and three for C18:0, explaining 33.6% and 56.7% of the total phenotypic variation observed respectively. One QTL each was detected for IV, C16:0 and C18:2. For the first time, this study has revealed QTLs associated with FAC in oil palm. The traits were largely controlled by a limited number of genomic regions with large effects. QTLs for five traits (IV, C14:0, C16:0, C18:0 and C18:1) were located in Group 1. All traits showed similar shaped LOD profiles suggesting that the same QTL is influencing all five traits. The fact that four of the traits are significantly correlated further supports this assumption. Furthermore, looking at the pathway for fatty acid biosynthesis where C16:0 is in fact elongated to C18:0 by the enzyme β-ketoacyl ACP synthase II (KASII), and C18:0 is subsequently desaturated by Δ9-stearoyl ACP desaturase to form C18:1, supports the fact that the same locus could be influencing these traits. Also considering that IV is a measure of unsaturation of oils and fats, C18:1 is the most abundant unsaturated fatty acid while C16:0 is the most abundant saturated fatty acid in palm oil, it is only logical to assume that the same genomic region is influencing these traits in oil palm.

QTLs for C14:0, C16:1, C18:0 and C18:1 were located on Group 15. The stearoyl ACP desaturase enzyme, although highly specific to the conversion of C18:0 to C18:1, is also known to sometimes act on C16:0 as a poor substrate and convert it to C16:1 [27]. This probably explains the strong negative correlation (r = -0.734) between C18:0 (stearic acid) and C16:1 (palmitoleic acid) and why the same QTL may be influencing the traits. As expected, the effect of the QTL for C16:1 and C18:0 is also in the opposite direction. The likelihood profile for the QTLs affecting the two traits in Group 15 (Figure 4) is also very similar, adding further strength to the argument that the same locus is influencing both traits.

Previously, a single preliminary QTL was reported for IV in oil palm in a similar population consisting of only 77 palms [39]. The LOD peak of 3.1 reported is not significant at the threshold level employed in the present study. Since no other similar work especially for FAC has been reported for oil palm, it was not possible to carry out a direct comparison with findings from other research groups. However, a comparison with other crops (mainly annual crops) is possible. For example, in maize, Alrefai *et al*. [40] detected 15 QTLs (in eight groups) associated with C16:0 only. Similarly, Mangolin *et al*. [41] detected 13 QTLs distributed in eight chromosomes for kernel oil content in maize. The low number of QTLs detected in this study, were however in agreement with the work by Somers *et al*. [42] and Jourdren *et al*. [43], who found that a few QTL loci could explain a large proportion of the phenotypic variation associated with one of the fatty acid components, C18:3 (linoleic acid) in *Brassica napus*. Furthermore, the same genomic region influencing two or more fatty acid components have also been reported for sunflower [44] and *Brassica napus *[45]. However, the experience in soybean was different where Panthee *et al*. [46] reported lack of common markers associated with C16:0, C18:0 and C18:1, although the same genomic region appears to be influencing the three 18-carbon unsaturated fatty acids (C18:1, C18:2 and C18:3). Nevertheless, it is important to note that the differences in QTLs mapped in this research cannot be directly compared to those reported above because of the different crop, type of markers, mapping population structure and the density of the genetic maps used in the analysis. The population size employed is another major factor that may explain differences in studies on QTL analysis, as the population size can affect the power to detect QTLs. The population size for QTL analysis in this study was 81 palms, smaller than that reported for some annual crops [40,46], which also makes direct comparison with other studies more difficult.

It is also noted that QTLs could not be detected for C18:3. The small population size employed limited power to detect QTLs of smaller effect. Analysis of further populations, particularly backcrosses derived from the same cross, may yield QTLs for this trait.

The QTLs identified in this study will provide breeders with a valuable tool to manipulate the FAC content in oil palm. For example, absence of the RFLP probe CB75A could be indicative of palms having oil with higher unsaturation level. The absence of the RFLP marker resulted in an increase of about 2.6% above the family mean for IV (level of unsaturation), and a decrease of about 6.5% below the family mean for C16:0 content (saturated fatty acid). If the marker/QTL linkage holds across different pedigrees, this RFLP marker could be used to enrich for genotypes with higher levels of unsaturation.

The association reported here was found only in a particular mapping population and as such may not yet be applicable for molecular breeding. Many researchers have pointed out that associations established in one cross may not hold true in other crosses [47,48]. However, Grattapaglia *et al*. [12] were of the opinion that substantial linkage disequilibrium can be maintained for marker/traits associations established in a single cross. The linkages established however can only be defined as "confirmed linkages" once they have been confirmed in a further sample, preferably by an independent group of investigators [49]. Nevertheless, it is heartening to note that QTLs for fatty acid composition have generally been validated across populations, even those associated with minor QTLs [29].

Although the efforts in Malaysia are largely directed towards decreasing levels of saturation, increasing levels of certain saturated fatty acids can also have some economic benefits. In this respect, there is interest in increasing the stearic acid content (C18:0), which can give rise to new applications such as cocoa butter substitution and personal care products (lotions, shaving creams and rubbing oils) [32]. This is also partly motivated by the substantial price differential between cocoa butter and commodity oils [50]. Like most plant oils, the oil palm has low stearate content of less than 5% [27]. The SSR marker P4T8 could play an important role in MAS for high stearate palms. The presence of the SSR alleles resulted in an increase of 6.8% above the family mean for C18:0 content.

An important point to note is that the saturated fatty acids, e.g. C16:0 and C18:0 are negatively correlated with total unsaturation (C18:1, C18:2 and C18:3) (data not shown). Furthermore, the QTLs for saturated and unsaturated fatty acids are largely in the opposite direction. As such, it is unlikely that a particular palm for high unsaturation and C18:0 can be bred. It may be more practical to select separately palms for high saturated and unsaturated oils.

Rajanaidu *et al*. [26] reported that repeatability of measurements for FAC is high indicating that a single measurement is sufficient to describe the fatty acid content of a bunch. Rajanaidu *et al*. [26] also predicted high heritability for most of the fatty acid traits in oil palm. Arasu *et al*. [33] reported that genotype × environment (G × E) interaction was not detected for any of the fatty acid traits in the 40 *E. guineensis *Nigerian germplasm populations analyzed. As such, good repeatability, high heritability and minimum G × E interaction suggest that FAC content is actually amenable to improvement with simple selection procedures. FAC can be improved rapidly using the strategy pointed out by Hospital *et al*. [51]. After having established the linkages, the genetic gain can be accelerated by scoring for markers associated with the QTLs for two generations without phenotypic observation. If the marker/QTL linkage holds true, Rance *et al*. [15] predicted that such a strategy could reduce the generation time by almost half for oil palm as the crosses can be made right after flowering (about 3 years), without having to wait for the fruits to be formed and analyzed (which can take up to 5 years).

## Conclusion

In this study, the QTLs were only detected for the male *E. guineensis *parent, T128. As such, we cannot conclude if the marker/QTL linkages will hold true for *E. oleifera*. Nevertheless, we believe that the linkages established between the marker and QTLs could be followed in backcross populations, which usually involve backcrossing the F_1 _hybrid to the *E. guineensis *parent. The QTLs identified in this study would also be potentially useful in exploiting the huge *E. guineensis *germplasm that Malaysia (through MPOB) has accumulated. QTLs with favorable alleles can be identified in the germplasm collection for incorporation into the existing breeding programmes. The high phenotypic variation explained by most of the QTL improves confidence in their application for MAS. Nevertheless, certain drawbacks should also be pointed out. There is always a possibility of linkage drag occurring, especially when involving germplasm collections and in oil palm interspecific hybrids, where unfavorable alleles such as that responsible for low yield are also incorporated together with the favorable alleles for higher unsaturation. However, as pointed out by Rance *et al*. [15], this can be minimized by selecting for QTLs with small confidence interval that defines a very narrow region.

## Methods

### Plant materials

An interspecific mapping population derived from the cross between *E. oleifera *palm UP1026 from Monteria, Colombia (female parent) and *E. guineensis *tenera palm T128 (male parent) from Nigeria was utilized in this study. Controlled self-pollination was adopted to generate the hybrids used in this mapping population. A total of 118 palms from this interspecific cross were planted and evaluated at one location at United Plantations, Teluk Intan, Perak, Malaysia.

### Preparation of genomic DNA

Leaf samples (young unopened or spear leaves) from all palms were collected and immediately frozen under liquid nitrogen and then stored at -80°C until DNA preparation could be carried out. DNA was prepared based on the method of Doyle and Doyle [52].

### Amplified fragment length polymorphism (AFLP) procedure

AFLP analysis was carried out by using the *Eco*RI/*Mse*I and *Taq*I/*Hin*dIII enzyme pairs. The *Eco*RI/*Mse*I assay was carried out by using the GIBCO BRL AFLP Analysis System 1 (Invitrogen, USA), essentially as described in the manufacturer's manual. The AFLP analysis using the *Taq*I/*Hin*dIII enzyme pairs was essentially performed as described by Rafalski *et al*. [53]. A subset comprising five samples (including the parents) was included in each electrophoresis run to ensure reproducibility.

### Restriction fragment length polymorphism (RFLP) analysis

#### i RFLP probes

The RFLP probes used to screen the interspecific hybrid mapping population were cDNA clones obtained from the various cDNA libraries (young etiolated seedlings, mesocarp, kernel and root) constructed previously as described by Cheah [54]. In addition, cDNA clones from a subtracted flower library [55] were also used to screen the mapping population. Plasmid DNA was prepared from individual clones and purified using column based kits (Qiagen, USA). The presence of the DNA insert was examined by restriction digestion (*Eco*RI) and electrophoresing on a 1.5% agarose gel. cDNA clones with insert sizes larger than 500 base-pairs (bp) were selected to screen for their ability to detect RFLP in the mapping population.

The DNA probes were diluted to a concentration of 5 ng/μl in TE buffer. The DNA probe (50 ng) was then labeled with α^32^P-dCTP (NEN^® ^Radiochemicals, Perkin Elmer, 3000 Ci/mmol stock) by using the Megaprime™ DNA Labeling system (GE Healthcare Life Sciences), as recommended by the manufacturer. The labeled probe was separated from the unincorporated nucleotides by purification through a Sephadex column as described in Sambrook *et al*. [56].

#### ii Southern blotting and hybridization

For the screening procedure, DNA samples (20 μg) from 10 palms (including the parental palms) were digested with 14 restriction enzymes (*Bam*HI, *Bcl*I, *Bgl*II, *Dra*I, *Eco*RI, *Hin*cII, *Hin*dIII, *Sca*I, *Sst*I, *Xba*I, *Bst*NI, *Hae*III, *Rsa*I and *Taq*I). The restricted DNA fragments were separated by electrophoresis in 0.9% agarose gel in 1× TAE (0.04 M Tris-acetate, pH 7.9, 1 mM EDTA pH 8.0) buffer and then transferred onto nylon membranes (Hybond N^+^, GE Healthcare Life Sciences) by vacuum blotting.

The 140 samples were then hybridized in turn with each candidate probe to identify the probe/restriction enzyme combination that gave a segregation profile. In the case of more than one enzyme showing polymorphism with a particular probe, the probe/enzyme combination that gave a clear single/low copy profile was selected for screening the entire mapping population. Replicate DNA preparations of the ten samples selected for screening (representing 8.7% of all the samples) were also screened concurrently with the entire mapping population. This was to facilitate reproducibility of RFLP profiles for different batches of DNA extraction. A subset comprising five samples (including the parental palms) was included as positive controls in every electrophoresis run to ensure reproducibility of the RFLP analysis.

Pre-hybridization and hybridization were carried out in glass tubes in a rotisserie oven at 65°C, essentially as described by Rahimah *et al*. [57].

### DNA sequencing and analysis

Plasmid DNA containing the cDNA clones was prepared as described above. cDNA inserts were sequenced from the 5' end with SK primer using the ABI PRISM™ Ready Reaction BigDye™ Terminator Cycle Sequencing Kit (Applied Biosystems, USA). Sequencing was performed on an ABI377 automated DNA sequencer (Applied Biosystems, USA). Raw ABI formatted chromatograms were base called using PHRED [58]. Customized Perl scripts were used to trim vector, adaptor, poly A-ends and low quality sequences. The edited sequences were searched against GenBank's non-redundant protein database using BLASTX [59]. Sequence similarities identified by BLASTX were considered statistically significant at a Poisson P value of ≤ 10^-10^.

### Microsatellites

#### i Isolation of microsatellites in oil palm

Degenerate primers described by Fisher *et al*. [60] and Brachet *et al*. [61] were used to isolate clones containing microsatellite sequences from oil palm. PCR was performed separately for the two parental DNA samples, T128 (Nigerian *E*. *guineensis*) and UP1026 (Colombian *E*. *oleifera*), using the protocol described by Fisher *et al*. [60]. The post PCR mix was cloned using the TOPO-TA cloning kit (Invitrogen, USA), essentially as recommended by the manufacturer. Sequencing was carried out on both strands using M13 forward and reverse primers (Invitrogen, USA) using an ABI377 sequencer (Applied Biosystems, USA). Specific primers in the flanking regions of the microsatellites were designed using the PRIMER3 software [62].

One primer for each primer pair was 5' end labeled at 37°C for 30 min using T4 polynucleotide kinase (Invitrogen, USA). The labeling reactions contained 50 pmoles of primer, 3 μl of γ-^33^P dATP (NEN^® ^Radiochemicals, Perkin Elmer, 3000 Ci/mmol), 1 U of T4 polynucleotide kinase in a total volume of 25 μl. Subsequently, the PCR reaction was carried out essentially as described by Billotte *et al*. [18]. After the PCR was completed, the reactions were stopped by the addition of 25 μl formamide buffer (0.3% bromophenol blue, 0.3% xylene cyanol, 10 mM EDTA pH 8.0, 97.5% deionized formamide). Each PCR reaction was subjected to electrophoresis on a 6% denaturing acrylamide gel containing 7 M urea using 0.5× TBE buffer at constant power of 40 W for 3 hours. The gels were then dried and exposed to X-ray film (Kodak) for 3 – 4 days at -80°C. Sizing of each allele was done using AFLP molecular weight ladder (Invitrogen, USA). To ensure reproducibility in the PCR reactions and electrophoresis runs, a subset of five samples (including the parents) was included in each batch of amplification reactions and subsequently included in each electrophoresis run.

#### ii Application of published oil palm microsatellite sequences

The 20 published single-locus microsatellite primer pairs [18] and the five EST-SSRs described by Chua *et al*. [63] were also tested on the mapping population. All the primer pairs were synthesized based on the published sequences and tested on a small number of individuals from the mapping population as described above. The informative primer pairs were then used to screen the entire mapping population.

In RFLP and SSR analysis, multiple loci detected by a single probe or primer pair were coded with the probe/primer name plus the suffix "I" or "II".

### Data analysis

The molecular results were analyzed according to the two-way pseudo-testcross approach described by Grattapaglia and Sederoff [34]. Data from RFLP, AFLP and SSR were scored and coded according to Lespinasse *et al*. [64] and Billotte *et al*. [13] (Table 2). Segregation ratios of markers were evaluated using the chi-square test for goodness-of-fit to the expected ratio (P < 0.05).

### Map construction

Map construction was carried out using *JoinMap *version 4.0 [65]. The interspecific cross was analyzed as a population resulting from a cross between two heterozygous diploid parents. The data of the female parent and the male parent were analyzed separately using the population type code "Cross Pollinator (CP)". Markers showing very significant distortion (P < 0.0001) were removed from the analysis. Subsequently, markers with 12 or more missing data points (approximately 10% or more missing genotypes) were also removed from the analysis, as the maximum likelihood mapping algorithm initially employed for map construction may be sensitive to having many unknown genotypes in the dataset [65].

The grouping of markers into linkage groups was evaluated using both independence LOD and recombination frequency. The maximum likelihood mapping (MLM) algorithm (using default parameters) was used to order the markers in the respective groups. Map order was improved by maintaining markers exhibiting a nearest neighbor stress value less than 2 cM. Plausible positions were determined to check the stability of map positions. In order to improve the map order, the total number of recombinations for each palm across linkage groups was also evaluated. The map order was further confirmed using Regression mapping (default parameters, recombination frequency < 0.4, LOD > 1 and jump = 5). Map distances were calculated by using the Haldane map function. In this study, the map order produced using MLM is presented.

### Quantitative data analysis

Quantitative traits associated with oil quality were of interest in this study. The criteria used to determine a ripe bunch was based on the standard practice in the industry of a minimum of 10 abscised fruitlets per bunch after harvest (irrespective of palm height) [1]. The harvested bunches were carefully tagged individually and bagged separately before being sent to the laboratory. Care was taken to prevent damaging the fruits when chopping the bunch and the oil was extracted, dried and filtered using the procedure described by Sharma [1]. The oil samples were then stored in UV-proof glass vials and sealed with Whatman film before being stored at -20°C. The samples were then sent to MPOB's analytical laboratory for the analysis of iodine value (IV), as well as the various fatty acid components i.e. myristic acid (C14:0), palmitic acid (C16: 0), palmitoleic acid (C16:1), stearic acid (C18:0), oleic acid (C18:1), linoleic acid (C18:2) and linolenic acid (C18:3). The analysis of IV and the individual fatty acid components was carried out essentially as described by Lin *et al*. [66].

Sampling was attempted on all 118 palms in the mapping population. However, owing to the variable degree of female sterility arising out of the interspecific nature of the cross, it was only possible to get sufficient fruit samples from 81 palms despite attempting to collect the samples over a two-year period. All these 81 palms were included in the final map construction and not omitted for any reason. QTL mapping analysis was initially performed using interval mapping as implemented in MapQTL version 5.0 [30]. Estimates of QTL position were obtained at the point where the LOD score assumes its maximum. The markers closest to the QTL position were then used as cofactors in MQM (or also known as Composite Interval Mapping) analysis, also implemented using the same software. The genome wide empirical thresholds for QTL detection (P < 0.05) were estimated using the permutation test as implemented in MapQTL version 5.0 [30]. In the permutation test, the presence of QTL is estimated from 1000 permutations carried out for each trait. The non-parametric Kruskal-Wallis test was also performed as a procedure of the MapQTL programme version 5.0 [30], in order to detect association between the markers and traits individually.

## Authors' contributions

RS and RAR performed the RFLP, AFLP and SSR analysis of the mapping population. MS carried out the breeding of the interspecific hybrid cross and collection of data for QTL analysis. RS and JJ constructed the genetic map and carried out the QTL analysis. LCLO sequenced, prepared and maintained the RFLP cDNA clones. ETLL edited and formatted the SSR and RFLP cDNA sequences for *GenBank *submission. RS drafted the manuscript. SCC, SGT, JMP, MS and RS participated in the design of the study. SCC, SGT and JMP supervised and coordinated the study. JJ, MS, SCC, LCLO and SGT critically revised the manuscript. All authors read and approved the final manuscript.
